# Supplementary material for: A new perspective on population genetics: Deciphering the relationship between genetic variants and disease prevalence in Psoriasis
Source: PLoS One. 2026 Mar 13;21(3):e0344204. doi: 10.1371/journal.pone.0344204 (PMC12987435; doi:10.1371/journal.pone.0344204)
Supplement: S1 File — (DOCX) [file pone.0344204.s037.docx]

**2.** **Computer program simulation**

In the former chapter, we have proved theoretically there is a matrix form linear correlation between the prevalence rate and the frequency of pathogenic variants. However, in reality, it is difficult for us to testify this hypothesis, for it is difficult to identify each pathogenic combination that 3 genotype combinations were extracted from. That is to say, it is impossible to define each term within that formula. But on the contrary, it is very easy to check whether there is a linear correlation between the average (median) of all the 3 genotype combination frequencies and the prevalence rate. So, an alternative solution is proposed: we need to investigate theoretically when there is a matrix form linear correlation exist, what is the probability of a linear correlation between the average (median) frequencies and the prevalence rate?

Nevertheless, even with the assistance of computer programs, the real pathogenicity combinations of psoriasis may be built up by four, five, six, or even more numbers, and some types could probably emerge multiple times. So, it is impossible to detect all the possibility (we don’t know how many real combinations, and how many genotypes each combination consisted of, etc.), but we do possible to investigate one specific case that may help us to comprehend the reality. In these simulation programs, we only considered 3 types pathogenic genotype combinations which are specific as 4 (a1*a2*a3*a4), 5 (b1*b2*b3*b4*b5), and 6 (c1*c2*c3*c4*c5*c6) genotype combinations to reduce the computation cost. To do this, we established four different models to detect whether there was a linear correlation existed between the average frequency of elements in vectors $A_{i}$ and P.

**2.1 Complete random model**

First, the complete random model, 15 random numbers were generated for three pathogenic combinations (a1*a2*a3*a4, b1*b2*b3*b4*b5, and c1*c2*c3*c4*c5*c6) by computer once a time for each population, and the linear least-square regression was used to detect whether there is a significant correlation between mean (median) of 3 genotype combinations and the prevalence.

e.g.

When we perform this command: ***python completely_random_time.py.txt 1000 20 1***, The first parameter means to perform linear correlation test 1000 times; the second means each time 20 different populations were generated; and the third means each pathogenic combination (4, 5, 6) turns up once. This program will generate 9 files which included the first (0.origin.txt, 0.txt), 10th (9.origin.txt, 9.txt), 100th (99.origin.txt, 99.txt), 1000th (999.origin.txt, 999.txt) permutations, and the final statistical results of 1000 permutations (result.txt).

The details of the first permutation and the results of 1000 permutation are showed in **S28 Table**. The first sheet includes three tables: 15 random numbers within three pathogenic combinations fluctuated in 20 populations (get from 0.origin.txt); 3 genotype combinations frequency obtained from 3 pathogenic combinations for each independent population (0.origin.txt); and the prevalence (P), Mean (X_bar) and median (X_md) of 3 genotype combinations frequency in 20 populations (0.txt). The next two sheets are linear regression analysis between the 3 genotype combinations frequency (mean and median) and prevalence in 1000 permutation tests (result.txt).

**2.2 Constrained model**

Considering there is gene flow between different populations, the frequency of disease-specific genotype in different ethnicities won't fluctuate sharply. Therefore, we regarded 15 random numbers generated for the first population as the reference frequency and limited the variation of corresponding random numbers for the other populations from the reference to 20% at most (the proportion can be changed in the program).

e.g.

When we perform this command: ***python convergence_loss_times_origion.py.txt 1000 20 20 0 0 1***, the first parameter means to perform linear correlation test 1000 times; the second means each time 20 different populations were generated; and the third means limited the variation of corresponding random numbers to the first generated population for the other populations to 20% at most; the fourth means the lost proportion of 3 genotypes combinations obtained from the pathogenic combinations is 0%; the fifth means 0% false 3 genotypes combinations mixing in; and the last means each pathogenic combination (4, 5, 6) turns up once too. This program will also generate 9 files which included the first (0.origin.txt, 0.txt), 10th (9.origin.txt, 9.txt), 100th (99.origin.txt, 99.txt), 1000th (999.origin.txt, 999.txt) permutations, and the final statistical results of 1000 permutations (result.txt).

The details of the first permutation and the results of 1000 permutation are showed in **S29 Table**.

**2.3 Constrained with lost model**

Consider the pathogenic genotype combinations are constructed by 4, 5 and 6 genotypes, but there are 3 genotypes combinations obtained by the CGCP program. Therefore, some of these combinations may be filtered by the control cohort despite they are extracted from the pathogenic genotype combinations. In addition, since the 3 genotypes combinations were obtained from one population by CGCP program, so, obviously, to investigate the relationship between prevalence and the frequency of these 3 genotypes combinations, the other populations should also be these corresponding combinations. That means, when the first population lost some combinations (for the follow example, in the first permutation, the first population lost a2a3a4, b1b2b5, etc.), the other populations must lost the same combinations.

e.g.

When we perform this command: ***python convergence_loss_times_origion.py.txt 1000 20 20 30 0 1***, the first parameter means to perform linear correlation test 1000 times; the second means each time 20 different populations were generated; and the third means limited the variation of corresponding random numbers to the first generated population for the other populations to 20% at most; the fourth means the lost proportion of 3 genotypes combinations obtained from the real combinations is 30%; the fifth means 0% fake combinations mixing in; and the last means each real combination (4, 5, 6) turns up once too. This program will also generate 9 files which included the first (0.origin.txt, 0.txt), 10th (9.origin.txt, 9.txt), 100th (99.origin.txt, 99.txt), 1000th (999.origin.txt, 999.txt) permutations, and the final statistical results of 1000 permutations (result.txt).

In the constrained model (previous section), the program generated $C_{4}^{3}$, $C_{5}^{3}$,$C_{6}^{3}$ combinations for 3 pathogenic combinations respectively.

The prevalence will be:

*P* =$\left[ \begin{matrix} f_{4} \\ f_{5} \\ f_{6} \end{matrix} \right]$= $\left[ \begin{matrix} a1a2a3a4 \\ b1b2b3b4b5 \\ c1c2c3c4c5 \end{matrix} \right]$

And the formula will look like this:

A × $A^{\#}$ + B × $B^{\#}$ + C × $C^{\#}$ = $\left[ \begin{matrix} C_{4}^{3} & C_{5}^{3} & C_{6}^{3} \end{matrix} \right]$ ×$\left[ \begin{matrix} f_{4} \\ f_{5} \\ f_{6} \end{matrix} \right]$= $\left[ \begin{matrix} C_{4}^{3} & C_{5}^{3} & C_{6}^{3} \end{matrix} \right]$ × P

However, in this constrained and lost model, for example, in the first permutation (**S30 Table)**, since the first population randomly lost 30% 3 genotypes combinations, and these combinations were also excluded in the other populations followed with the first population.

The 3 genotypes combinations and the residual terms in the first permutation are:

A=$\left[ \begin{matrix} a1a2a3 & a1a2a4 & a1a3a4 \end{matrix} \right]$

$A^{\#}$= $\left[ \begin{matrix} a4 \\ a3 \\ a2 \end{matrix} \right]$

B= $\left[ \begin{matrix} \begin{matrix} b1b2b3 & b1b2b4 & b1b3b4 \end{matrix} & \begin{matrix} b2b3b4 & b2b3b5 & \begin{matrix} b2b4b5 & b3b4b5 \end{matrix} \end{matrix} \end{matrix} \right]$

$B^{\#}$= $\left[ \begin{matrix} \begin{matrix} b4b5 \\ b3b5 \\ b2b5 \end{matrix} \\ \begin{matrix} b1b5 \\ b1b4 \\ \begin{matrix} b1b3 \\ b1b2 \end{matrix} \end{matrix} \end{matrix} \right]$

The detail of matrix C and $C^{\#}$are not shown here.

So, the formula will change to this:

A × $A^{\#}$ + B × $B^{\#}$ + C × $C^{\#}$ = $\left[ \begin{matrix} 3 & 7 & 13 \end{matrix} \right]$ ×$\left[ \begin{matrix} f_{4} \\ f_{5} \\ f_{6} \end{matrix} \right]$= $\left[ \begin{matrix} 3 & 7 & 13 \end{matrix} \right]$ × P

The ultimate result is that the constant vector C changes from $\left[ \begin{matrix} C_{4}^{3} & C_{5}^{3} & C_{6}^{3} \end{matrix} \right]$ to$\left[ \begin{matrix} 3 & 7 & 13 \end{matrix} \right]$ . It means in this model, elements in vector C may less than $C_{k}^{3}$ when some combinations were filtered. Nonetheless, the formula is still presenting linear correlation in matrix form.

**2.4 Constrained with lost and mixed model**

Due to the control group is not large enough, there might be some interfused fake combinations that are not extracted from the pathogenic genotypes combinations, but are still output by CGCP method which may cause type-I error. In this constrained with lost and mixed model, we added another parameter which is the mix rate and to investigate the relationship between the mean (median) of these combination frequency and the prevalence.

e.g.

When we perform this command: ***python convergence_loss_times_origion.py.txt 1000 20 20 30 10 1***, the first parameter means to perform linear correlation test 1000 times; the second means each time 20 different populations were generated; and the third means limited the variation of corresponding random numbers to the first generated population for the other populations to 20% at most; the fourth means the lost proportion of 3 genotypes combinations obtained from the real combinations is 30%; the fifth means 10% fake combinations mixing in; and the last means each real combination (4, 5, 6) turns up once too. This program will also generate 9 files which included the first (0.origin.txt, 0.txt), 10th (9.origin.txt, 9.txt), 100th (99.origin.txt, 99.txt), 1000th (999.origin.txt, 999.txt) permutations, and the final statistical results of 1000 permutations (result.txt).

Doesn’t like former three models, since there are some fake combinations mix in, this model is not coincidence with the formula. However, for this model is closer to the real world, it is interesting to see how much has been changed when compare with the previous models.

The details of the first permutation and the results of 1000 permutation are showed in **S31 Table.**
