# Supplementary material for: A new perspective on population genetics: Deciphering the relationship between genetic variants and disease prevalence in Psoriasis
Source: PLoS One. 2026 Mar 13;21(3):e0344204. doi: 10.1371/journal.pone.0344204 (PMC12987435; doi:10.1371/journal.pone.0344204)
Supplement: S2 File — (DOCX) [file pone.0344204.s038.docx]

**Mathematical Inference:**

**A Linear Correlation Exist in Matrix Form between the Prevalence Rate and the Frequency of Pathogenic Variants**

It was obvious that if psoriasis is caused by one unique four genotype combination and the frequency of each genotype was defined as a_1_, a_2_, a_3_, a_4,_ the prevalence will equal the frequency of this four genotypes combination (*P* = a_1*_a_2*_a_3*_a_4_, when each variant was independent of each other as described by CGCP method). The CGCP method in this study will get up to 4 different combinations of 3 variants when all outcomes came from the real one and have not been filtered. Finally, the combinations can be defined as a vector with $C_{4}^{3}$ elements: A=$\left[ \begin{matrix} {a_{1}a_{2}a}_{3} & {a_{1}a_{2}a}_{4} & {a_{1}a_{3}a}_{4} & {a_{2}a}_{3}a_{4} \end{matrix} \right]$, and the frequency of the residual genotype can also make up another vector with $C_{4}^{1}$ elements:$A^{\#}$ =$\left[ \begin{matrix} a_{4} \\ a_{3} \\ a_{2} \\ a_{1} \end{matrix} \right]$; then, A × $A^{\#}$ will equal to $C_{4}^{3}$*P*.

While the prevalence of psoriasis was made up by 3 different combinations that consisted of 4, 5 and 6 distinct genotypes and the combined frequency can be defined as *f*_4_ (the frequency of each genotype was defined as a_1_, a_2_, a_3_, a_4_), *f*_5_ (the frequency of each genotype was defined as *b*_1_, *b*_2_, *b*_3_, *b*_4_, *b*_5_) and *f*_6_ (the frequency of each genotype was defined as *c*_1_, *c*_2_, *c*_3_, *c*_4_, *c*_5_, *c*_6_) respectively, we can assume the prevalence of psoriasis as:

*P* = *f*_4_+*f*_5_+*f*_6_=*a*_1_**a*_2_**a*_3_**a*_4_+*b*_1_**b*_2_**b*_3_**b*_4_**b*_5_+*c*_1_**c*_2_**c*_3_**c*_4_**c*_5_**c*_6_;

If this prevalence is set as a vector form: *P* =$\left[ \begin{matrix} f_{4} \\ f_{5} \\ f_{6} \end{matrix} \right]$ , then we obtain the similar formula as:

A × $A^{\#}$ + B × $B^{\#}$ + C × $C^{\#}$ = $\left[ \begin{matrix} C_{4}^{3} & C_{5}^{3} & C_{6}^{3} \end{matrix} \right]$ ×$\left[ \begin{matrix} f_{4} \\ f_{5} \\ f_{6} \end{matrix} \right]$= $\left[ \begin{matrix} C_{4}^{3} & C_{5}^{3} & C_{6}^{3} \end{matrix} \right]$ × P

Finally, in the general model, we set the prevalence in the population of a given ethnicity as P:

P =$\left[ \begin{matrix} f_{1} \\ f_{2} \\ \ldots\\ f_{n} \end{matrix} \right]$;

Where *n* is the total number of real pathogenic combinations that these 3 variant combinations extracted from, and *f_i_* (i from 1 to n) is the frequency of one real causal combination.

Then, if all the real combination numbers are more than 3 and each of the 3 genotype combinations extracted from the real combinations was obtained by the CGCP method, we defined vector C = $\left[ \begin{matrix} C_{4}^{3}\ldots& C_{5}^{3}\ldots& C_{6}^{3} \end{matrix}\ldots C_{k}^{3}\ldots\right]$ (each element of vector C is determined by the real combination number of causal variants; K is an integer starting from 4 which can be discontinuous. The total of vector elements is n). Then, the observed frequency of the 3 variant combinations in the population of a given ethnicity will satisfy this formula:

$\sum_{i=1}^{n} A_{i}\times A_{i}^{\#}$ = C× P;

In this formula, when psoriasis consists of several combinations, as described by our method, and most 3 genotype combinations extracted from real combinations are obtained, then the number of elements in vectors $A_{i}$ and $A_{i}^{\#}$ will be fixed in different ethnicities (even if some outcomes came from the real one have been filtered, the correspondent element of vector C will be less than $C_{k}^{3}$, but it also will be fixed in different ethnicities), which means vector C will fixed as a constant although it was not possible to confirm how many genotypes consisted of the real combination for each observed 3 genotype combination. In this study, the goal is to investigate the relationship between the observed frequency and prevalence in different ethnicities, which means, in this formula, is to investigate the relationship between vector $A_{i}$ and P.
