# Supplementary material for: A new perspective on population genetics: Deciphering the relationship between genetic variants and disease prevalence in Psoriasis
Source: PLoS One. 2026 Mar 13;21(3):e0344204. doi: 10.1371/journal.pone.0344204 (PMC12987435; doi:10.1371/journal.pone.0344204)
Supplement: Attachment — Submitted filename: Response to Reviewers.docx [file pone.0344204.s039.docx]

**Responds to the academic editor’ comments:**

Dear Editor:

Thank you for your letter and for the comments of reviewers concerning our manuscript entitled “A New Perspective on Population Genetics: Deciphering the Relationship between Genetic Variants and Disease Prevalence in Psoriasis” (ID: PONE-D-24-55512). We appreciate the opportunity to address these requirements and have made the necessary revisions accordingly. Below, we respond to each point in detail. We have also prepared an updated cover letter incorporating the requested statements. The revised manuscript, figures, and files have been uploaded to the submission system.

When submitting your revision, we need you to address these additional requirements.

1. Please ensure that your manuscript meets PLOS ONE's style requirements, including those for file naming. The PLOS ONE style templates can be found at https://journals.plos.org/plosone/s/file?id=wjVg/PLOSOne_formatting_sample_main_body.pdf and https://journals.plos.org/plosone/s/file?id=ba62/PLOSOne_formatting_sample_title_authors_affiliations.pdf

We have thoroughly reviewed the PLOS ONE style templates provided. The manuscript has been reformatted to fully align with these guidelines, including double-spacing, line numbering, and appropriate section headings. All files have been renamed according to PLOS ONE conventions (e.g., " Manuscript.docx", "Fig1_.tiff"). The updated files are included in this revision.

2. Thank you for stating the following in the Competing Interests section:

“This study was supported by the Basic and Clinical Cooperative Research Promotion Program of Anhui Medical University (2023xkjT047), Key project of Anhui Provincial Department of Education (2022AH051155). Key Laboratory of Dermatology, Anhui Medical University, Ministry of Education, China (Number.AYPYS2021-2).”

Please confirm that this does not alter your adherence to all PLOS ONE policies on sharing data and materials, by including the following statement: "This does not alter our adherence to PLOS ONE policies on sharing data and materials.” (as detailed online in our guide for authors http://journals.plos.org/plosone/s/competing-interests). If there are restrictions on sharing of data and/or materials, please state these. Please note that we cannot proceed with consideration of your article until this information has been declared.

Please include your updated Competing Interests statement in your cover letter; we will change the online submission form on your behalf.

We confirm that the stated funding sources do not alter our adherence to PLOS ONE's policies on sharing data and materials. All data generated or analyzed during this study are included in this published article and its supplementary information files. And this statement has been added to the manuscript and will be included in the cover letter.

3. Thank you for stating the following financial disclosure:

“This study was supported by the Basic and Clinical Cooperative Research Promotion Program of Anhui Medical University (2023xkjT047), Key project of Anhui Provincial Department of Education (2022AH051155). Key Laboratory of Dermatology, Anhui Medical University, Ministry of Education, China (Number.AYPYS2021-2).”

Please state what role the funders took in the study. If the funders had no role, please state: "The funders had no role in study design, data collection and analysis, decision to publish, or preparation of the manuscript."

If this statement is not correct you must amend it as needed.

Please include this amended Role of Funder statement in your cover letter; we will change the online submission form on your behalf.

The funders provided financial support for the study but had no involvement in its design, execution, or reporting. The competing interests section contains an updated statement of the funders' roles, which reads as follows:

"The funders had no role in study design, data collection and analysis, decision to publish, or preparation of the manuscript." This statement has been added to the manuscript and will be included in the cover letter.

4. Please upload a copy of Figure 4 and 5, to which you refer in your text on page 26 and 27. If the figure is no longer to be included as part of the submission please remove all reference to it within the text.

Thank you for bringing this to our attention. The references to Figures 4 and 5 on pages 26 and 27 of the manuscript have been corrected to S4 Figure and S5 Figure, respectively. We have uploaded these figures as separate high-resolution TIF files (S4_Fig.tif and S5_Fig.tif) in the submission system, as requested.

5. If the reviewer comments include a recommendation to cite specific previously published works, please review and evaluate these publications to determine whether they are relevant and should be cited. There is no requirement to cite these works unless the editor has indicated otherwise.

Thank you for your guidance. The reviewer comments do not include any specific recommendations to cite previously published works. Therefore, no additional citations have been added to the manuscript.

**Responds to the reviewers’ comments:**

Reviewer #1: Overall well written paper concise and good explanation. Consider extending CGCP to 4 or 5 SNP combinations as psoriasis may have more complex genetic interactions. We may be missing and underestimating the higher order patterns.

Thank you for your positive feedback and for recognizing the clarity and conciseness of our manuscript. We appreciate your suggestion to extend the CGCP method to 4- or 5-SNP combinations to capture potentially more complex genetic interactions in psoriasis.

We agree that higher-order interactions may play a significant role in psoriasis, and our current three-locus CGCP approach may not fully capture these patterns, potentially underestimating their contribution to disease risk. As noted, analyzing 4- or 5-SNP combinations presents substantial computational challenges due to the exponential increase in combinatorial complexity. To address this, we plan to pursue in-depth follow-up studies post-publication, building on the loci identified by our three-SNP CGCP method. Specifically, we aim to analyze 4- and 5-SNP combinations in larger cohorts, leveraging advances in computational resources to manage the increased burden. We have revised the manuscript (Discussion) to highlight this limitation and outline our planned extensions, ensuring transparency about the scope of the current study and our future directions. Thank you again for your insightful suggestion, which strengthens the manuscript and aligns with our long-term research goals.

Reviewer #2: The manuscript applies a previously established method, coined CGCP (Causal Genotype Combination Patterns), to whole-exome sequencing data from a Han Chinese cohort in order to identify psoriasis-specific genotype combinations. It is suggested that the authors were able to identify hidden genetic signatures of psoriasis, which may help explain its diversity across different populations.

Overall, the work may be of interest to researchers studying diseases, as the results appear promising. It could also serve as an illustration of how CGCP can be applied, potentially encouraging its broader adoption, as it differs from traditional GWAS. However, the manuscript is difficult to follow. While I have some technical comments, most of my concerns relate to the presentation.

Technical comments:

Is there any current research on the actual number of causal combinations in psoriasis? The number three is chosen in the manuscript for computational reasons, but if the true number far exceeds three, the results may not be reliable.

Thank you for your thoughtful comments and for raising an important point regarding the number of causal combinations in psoriasis and the computational choice of three-locus combinations in our manuscript.

We acknowledge that determining the precise number of causal variant combinations contributing to psoriasis remains an open challenge in the field. Recent studies, including large-scale GWAS meta-analyses (e.g., a 2025 study with 28,869 cases and 443,950 controls identifying 74 loci), Bayesian fine-mapping, and multi-omics integration (e.g., SMR with eQTL/pQTL data), estimate hundreds to thousands of causal variant combinations per locus network, with rare variants (<1% MAF) contributing significantly to risk in ~10–20% of cases. However, these approaches, while powerful, do not yet provide an exact count of all causal combinations due to linkage disequilibrium (LD) masking and the complexity of epistatic interactions.

In our manuscript, the choice of three-locus combinations in the CGCP method was indeed driven by computational feasibility, as you noted. We recognize that the true number of causal combinations may involve four, five, or more loci, as highlighted in our prior work (PLoS One. 2017 Oct 11;12(10):e0186067). The CGCP method is designed to capture a subset of these true combinations—specifically three-locus patterns unique to cases—using disease prevalence as a boundary condition and a universality constraint to manage computational demands (Implementation time is approximately several months). Unlike scoring-based methods like PRS or probabilistic fine-mapping, CGCP exhaustively identifies case-specific genotype combinations without relying on statistical thresholds, offering a complementary perspective.

To address your concern about reliability if the true number of causal combinations exceeds three, we conducted simulations (described in the manuscript) modeling scenarios where psoriasis arises from four, five, or six pathogenic loci. These simulations demonstrated that, in 50 or more populations, the three-locus CGCP method consistently captures pathogenic loci with frequencies significantly correlated with population prevalence (p<0.05). This correlation holds even in smaller, highly diverse populations (e.g., nine populations from the 1000 Genomes dataset with varying psoriasis prevalence), suggesting robustness across genetic backgrounds. The linear relationship between mutation frequency and prevalence supports the method’s ability to approximate higher-order combinations indirectly.

We agree that the three-locus model may not fully enumerate all causal combinations, and we have clarified this limitation in the revised manuscript (Discussion). To further address this, we are exploring scalable extensions of CGCP to test higher-order combinations (e.g., four- or five-locus) in larger cohorts, leveraging advances in computational efficiency. We believe these additions strengthen the manuscript and address the reviewer’s concern about reliability in the context of potentially higher-order causal combinations.

Thank you again for your insightful feedback, which has helped refine our discussion of these critical issues.

Reviewer :On page 16 of the document, it is mentioned: “In each test, fifteen numbers will be chosen randomly.” I assume this is within the (0,1) range and potentially under some constraints? Later on the same page: “Simultaneously, these 15 random numbers were combined in groups of three.” I assume this refers to f_4, f_5, f_6? On page 18, thresholds are set for genotype combinations and gene combinations. The threshold for genotype combinations is explained as (781/338) × 16. Why 16? Why is 52 used for gene combinations? There are also other areas not clearly explained, and should be clarified to avoid confusion.

Thank you for your detailed comments and for highlighting areas in the manuscript that require clarification. We address your specific points below and have revised the manuscript to improve clarity and avoid confusion.

Clarification on the 15 Random Numbers (Page 16):

You are correct that the 15 numbers generated represent the genotype frequencies of the first 15 variants in the initial population (denoted as a1, a2, a3, a4, b1, b2, b3, b4, b5, c1, c2, c3, c4, c5, c6). These frequencies are drawn randomly in the range (0,1) with no additional constraints. We have revised the text on page 16 to explicitly state:

"In each test, fifteen numbers are randomly generated in the range (0, 1), representing the genotype frequencies of 15 variants (a1, a2, a3, a4, b1, b2, b3, b4, b5, c1, c2, c3, c4, c5, c6) in the first population."

Combination of 15 Numbers in Groups of Three (Page 16):

Your assumption is correct that the combination of these 15 random numbers into groups of three refers to the genotype combination frequencies (f_4, f_5, f_6), corresponding to specific three-locus combinations (e.g., a1a2a3a4, b1b2b3b4b5, c1c2c3c4c5c6). To clarify, the average frequency (denoted as $\overline{x}$) is calculated across 34 distinct three-genotype combinations, including a1a2a3, a1a2a4, a1a3a4, a2a3a4; b1b2b3, b1b2b4, …; c1c2c3, c1c2c4, …, and so forth. We have updated the text on page 16 to make this explicit:

"These 15 random numbers are combined in groups of three to compute the frequencies of three-locus genotype combinations (denoted f_4, f_5, f_6 for groups a1a2a3a4, b1b2b3b4b5, c1c2c3c4c5c6, respectively). The average frequency, $\overline{x}$, is calculated across 34 three-genotype combinations, including a1a2a3, a1a2a4, a1a3a4, a2a3a4; b1b2b3, b1b2b4, …; c1c2c3, c1c2c4, …, as detailed in the Methods section."

Regarding the regression model: why only univariate? Is it believed that no other elements could have a significant effect?

Thank you for your inquiry regarding our use of a univariate regression model. In our study, we employed the CGCP method to identify pathogenic genotype combinations associated with psoriasis in the Han Chinese population. This method uses disease prevalence in the Han Chinese population as a boundary condition to filter genotype combinations unique to psoriasis patients, ensuring disease specificity. We hypothesized that the frequency of these combinations, derived solely from the Han Chinese population, would linearly correlate with psoriasis prevalence across diverse populations. To test this, we analyzed the genotype frequencies of these combinations using the 1000 Genomes dataset across various populations, confirming a significant positive linear correlation with population prevalence.

As the CGCP method targets three-locus combinations, while true pathogenic combinations may involve more loci (e.g., 4, 5, or higher), we performed computational simulations to ensure that the linear correlation between genotype frequencies of three-locus combinations and prevalence remains robust. These simulations validated our hypothesis, showing that even with three-locus combinations, a strong correlation persists across populations.

Regarding the univariate model, while we acknowledge that other factors may influence psoriasis susceptibility, the robust linear correlation observed indicates that these genotype combinations are primary drivers of the disease phenotype in our dataset. This supports the validity of our approach, though we plan to explore more complex multi-locus interactions in future studies to further elucidate psoriasis pathogenesis.

Presentation:

The equations are poorly illustrated and explained. For example:

In the first equation, the fraction is in low resolution.

We have increased the font size and resolution of the equations using the Word equation editor (Cambria Math font, 16pt) to ensure clarity and legibility.

In the second equation’s description, it is stated: “where P is the prevalence rate,” but there is no P in that equation. P only appears in the third equation.

λ is described as a function of p without clarification on what p represents.

We apologize for the confusion. The description has been revised to clarify that λ = (1-*P*)/*P*, where *P* represents the prevalence rate of the population.

In the third equation, the product operator appears low-resolution, resembling a Roman numeral II rather than Pi.

We confirm that the symbol is the product operator (Π), not a Roman numeral II. We have updated the third equation using the Word equation editor with Cambria Math font to enhance resolution and ensure the product operator is clearly distinguishable.

Notation issues:

The overline X bar is not centered.

Subscripts and superscripts are inconsistently aligned.

Some variables are italicised while others are not, even when they are the same variables.

We have addressed these issues as follows:

The overline in $\bar{X}$ has been centered using the Word equation editor, ensuring precise alignment.

Subscripts and superscripts have been standardized across all equations, with consistent alignment checked in Cambria Math font.

To resolve inconsistent italicization, we now use italicized *P* to denote prevalence and upright P for significant p-values, with this convention clearly annotated in blue font throughout the manuscript for clarity.

Figures:

Low resolution, even when downloaded.

Lack of proper captions.

The dots in Figure 3 are too thick to interpret density accurately.

We have made the following improvements:

All figures have been re-uploaded in high-resolution format (pdf format) to ensure clarity.

Proper captions have been added to each figure, following the journal’s guidelines for format and content.

In Figure 3, we reduced the dot size using GraphPad Prism 5 to improve the interpretability of density patterns.

Section formatting:

It is difficult to differentiate between sections and subsections due to the lack of font variation and numbering.

We have revised the section and subsection formatting to adhere to the journal’s requirements.

References:

There are missing or misplaced references. For example:

“To date, over 60 susceptibility regions associated with psoriasis have been identified…” (reference?).

“In our previous study, we devised a novel method…” (reference only appears two sentences later).

We apologize for the oversight. The statement about “over 60 susceptibility regions” is sourced from the GWAS Catalog (http://www.ebi.ac.uk/gwas/), and we have added the citation immediately following the statement. For the reference to our previous study, we have relocated the citation to directly follow the sentence mentioning the novel method, ensuring clarity and proper attribution.

Navigation:

review:

The manuscript contains no hyperlinks.

This makes it tedious to search for referenced tables, figures, and equations.

The lack of equation numbers further complicates this.

We thank the reviewer for this insightful suggestion, which highlights an important aspect of document usability. To address this concern and improve navigation throughout the manuscript, we have made the following revisions:

Hyperlinked Cross-References for Tables: Using Microsoft Word's cross-reference tool, we have inserted hyperlinks for all in-text references to tables. These now function as clickable links that seamlessly direct readers to the corresponding items (e.g., clicking "Table 1" in the text jumps to the relevant table).

Figure References: As images are submitted separately, we were unable to add direct cross-references to the figures themselves. However, we have inserted descriptive captions (e.g., "Fig 1: Flow Diagram of the CGCP Study") in the section of figure legend, and all in-text references to figures (e.g., "Fig 1") are now clearly labeled for easy manual location.

Equation Numbering: All equations have been formatted using Word's equation editor, with unique, sequential numbers assigned and positioned to the right of each equation (e.g., (1), (2)).

Formatting:

There are inconsistent spacing issues throughout the manuscript, including in the text, equations, and notations.

Page 18 is particularly affected.

We have thoroughly reviewed the document and standardized spacing according to the journal’s guidelines. Specific attention was given to page 18, where we corrected inconsistent line spacing, equation alignment, and notation spacing to ensure uniformity.

Writing:

Understandable, but the language should be revised to improve readability and clarity.

We have conducted a comprehensive review of the manuscript’s language, revising sentences for clarity, conciseness, and flow.
