# Supplementary material for: A new perspective on population genetics: Deciphering the relationship between genetic variants and disease prevalence in Psoriasis
Source: PLoS One. 2026 Mar 13;21(3):e0344204. doi: 10.1371/journal.pone.0344204 (PMC12987435; doi:10.1371/journal.pone.0344204)
Supplement: Attachment — Submitted filename: Response to Reviewers-R2.docx [file pone.0344204.s040.docx]

**Responds to the academic editor’ comments:**

Dear Editor:

Thank you for your letter and for the comments of reviewers concerning our manuscript entitled “A New Perspective on Population Genetics: Deciphering the Relationship between Genetic Variants and Disease Prevalence in Psoriasis” (ID: PONE-D-24-55512). We appreciate the opportunity to address these requirements and have made the necessary revisions accordingly. Below, we respond to each point in detail.The revised manuscript, figures, and files have been uploaded to the submission system.

Reviewer #2: The authors have responded to my concerns well; however, some of them have not been fully addressed.

The following comment remain unanswered:

On page 18, thresholds are set for genotype combinations and gene combinations. The threshold for genotype combinations is explained as (781/338) × 16. Why 16? Why is 52 used for gene combinations?

Thank you for your positive feedback. In the part of “The background noise of CGCPs” (line 211-215), We explained why 16 serves as the coefficient for calculating the genotype combinations.

“In the first scenario, CGCP analysis was performed on 338 “cases” and 338 controls, involving 482 SNPs, that showed significant differences between cases and controls after quality control (S5 Table). A total of 9,802 genotype combinations considered specific to psoriasis were identified, with the maximum number of specific combinations being 16 (4.7%) (S6 Table).”

Since the maximum number of genotype combinations in the background noise is 16, in order to reduce the influence of background noise on the actual number of combinations, we suppose that the actual number of genotype combinations should be at least greater than 16. Thus, we used 16 as the coefficient for calculating the genotype combinations.

In addition, in the part of “The background noise of CGCPs” (line 219-226), We explained why 52 serves as the threshold for calculating the gene combinations.

“In the second scenario, association analysis was conducted using 548,357 SNPs located outside psoriasis-associated regions, based on whole-exome sequencing data. After quality control and pruning, 570 SNPs were included in the CGCP analysis (S8 Table). A total of 63,657 genotype combinations were identified as specific to psoriasis, with the maximum observed number of specific combinations being 32 (4.1%) (S9 Table). After annotating the SNPs in these psoriasis-specific combinations to genes, 62,971 combinations of variants of genes were obtained, and the maximum number of specific combinations of variants of genes was 52 (6.5%) (S10 Table).

Based on the scale-up principle, thresholds for investigating background noise in psoriasis-specific genotype combinations and combinations of variants of genes were established. Specifically, the threshold for genotype combinations was set at 37, calculated as [(781/338) × 16], and that for combinations of variants of genes was set at 52. ”

Since the maximum number of specific gene combinations in the background noise is 52, in order to reduce the influence of background noise on the actual number of combinations, we suppose that the actual number of gene combinations should be at least greater than 52. Thus, we used 52 as the coefficient for calculating the gene combinations.

Regarding the regression model, could confidence interval for the coefficients be provided?

We sincerely thank the reviewer for this valuable suggestion.

In the revised manuscript, we now report the 95% confidence intervals for all regression coefficients together with adjusted R² values (page 3, lines 58).

Disease prevalence is now expressed as a proportion (0–1 scale) rather than as a percentage (0–100), consistent with standard practice in genetic epidemiology when both the outcome (prevalence) and the predictor (allele frequency) are bounded between 0 and 1. This rescaling explains the apparent ~100-fold increase in the magnitude of the slope relative to the original submission.

Ninety-five percent confidence intervals for the regression coefficients were calculated using the t-distribution with n−2 degrees of freedom (df = 7). Adjusted R² was computed as Adjusted R²= 1 − [(1 − R²)(n − 1)/(n − p)], where n = 9 (number of populations) and p = 2 (intercept + slope).

The updated linear regression models are:

Median allele frequency Prevalence = 61.72 × median allele frequency (95% CI for slope: 21.60 to 101.84) + 0.48, P = 8.31 ×10E-3 (R² = 0.654, adjusted R² = 0.605)

Mean allele frequency Prevalence = 47.63 × mean allele frequency (95% CI for slope: 11.25 to 84.01) + 0.62, P = 0.014 (R² = 0.571, adjusted R² = 0.510).

Thus, a 0.01 (i.e., 1 percentage point) increase in frequency of these psoriasis-specific genotype combinations is associated with an absolute increase in disease prevalence of approximately 0.48-0.62 percentage points across populations, and the association is statistically significant in both models.

We believe these revisions substantially improve the clarity, accuracy, and interpretability of the regression analyses.

Figures: They now have good resolution when downloaded but still appear blurry in the manuscript—Figure 2 is especially affected. Perhaps this is simply a compilation issue?

Thank you for pointing out the issue. We have re-uploaded Figure 2.

Notations and Formatting: Whilst there has been significant improvements, many inconsistencies remain. Below are some examples I noticed when glancing through the manuscript, though these are certainly not the only ones. The authors should carefully review the entire manuscript to eliminate any remaining inconsistencies.

Below equation (2), alpha_i and beta_i appear italicised, but are not in the equations. Their font also appear smaller compared to the previous lambda=(1-P)/P. F(gi) also appears differently in three places: above equation (2) (italicised), in equation (2) and in equation (3) (F and (gi) separated, different style of parentheses from equation (2).

Thank you for your guidance. We have already review carefully about our manuscript and the modified parts are highlighted with blue text.

On a related note, why is the population genotype frequency denoted as F(gi)? Currently on first glance it appear to be a function notation. Unless there are specific reason behind this, wouldn't F_i or something similar be more standard?

Thank you for your guidance. We modified the denotation of population genotype frequency from *F(gi)* to *F_i*.

Between lines 180 and 193, there are several notation inconsistencies regarding a_1,f_4 etc. Some have subscripts, others do not; font sizes also vary.

Thank you for your kind reminder. We have standardized the formats of all the fonts and symbols in the manuscript.

Whether there is a spacing before references. (lines 403, 405)

Thank you for your kind reminder. We have already delete the spacing.

Decimals, some are written as 0.x, some .x.

In equations multiplications, some are written as x*y, the others x * y.

Thank you for your kind reminder. We have standardized the formats of all the fonts and symbols in the manuscript.

Reviewer #3: Zheng and coworkers present a well-written follow-up study on their recently established “Causal Genotype Combination Patterns” (CGCP) method to investigate large samples for specific combinations of gene variants, i. e., genotypes. By applying this method to psoriasis, a common skin disorder, they were able to identify numerous genotype combinations, converging into 134 candidate genes, most of which are novel candidates. The manuscript has been reviewed by experts before, and the authors apparently addressed their questions and remarks adequately.

I find this manuscript quite informative, and I have some recommendations to improve the paper:

- Introduction, line 82: “The gradually increasing prevalence…” – This sentence is quite vague, speculative, and should be referenced at best.

Thank you for bringing this to our attention. We have added the corresponding references (No.8 and 9).

- Materials and Methods, line 116: The company and location for the array used should be mentioned here.

Thanks for your advice. We have provided information about the company and location of the array.

- Lines 153 to 167 (“background noise”), and lines 220 to 232 (also“background noise”): These paragraphs appear in duplicate, slightly differing from each other, and should be merged into one, preferentially with all numbers mentioned therein.

Thank you for your thoughtful comments. In order to maintain the conventional structure of a research paper, we chose not to directly integrate the Methods section (lines 153-167) with the Results section (lines 220-232). However, we have given careful consideration to your suggestions, overlapping content between the two sections has been streamlined to avoid redundancy and enhance readability. Corresponding revisions have been made in the manuscript.

Line150-159 (Previously it was line 153 - 167)

The background noise of CGCPs

To evaluate the background noise in CGCP calculations, we performed analyses using samples and SNPs unrelated to psoriasis. First, the 676 healthy controls were randomly divided into two groups, with one group designated as “patients.” CGCP analysis was then conducted using variants selected from psoriasis-associated loci. Additionally, in an independent dataset comprising 781 cases and 676 controls, CGCP analysis was performed using SNPs located outside the psoriasis-associated regions, thereby capturing background noise originating from SNPs unrelated to psoriasis. The threshold for background noise was determined by comparing the maximum number of “specific” combinations derived from these two unrelated datasets (Fig 1).

Line210-228 (Previously it was line 220 - 232)

Investigation of Background Noise

To evaluate the background noise in CGCP calculations, we performed analyses using samples and SNPs unrelated to psoriasis. First, the 676 healthy controls were randomly divided into two groups, with one group designated as “patients.” CGCP analysis was then conducted using variants selected from psoriasis-associated loci. Additionally, in an independent dataset comprising 781 cases and 676 controls, CGCP analysis was performed using SNPs located outside the psoriasis-associated regions, thereby capturing background noise originating from SNPs unrelated to psoriasis. The threshold for background noise was determined by comparing the maximum number of “specific” combinations derived from these two unrelated datasets.

The specific results were as follows: In the first scenario, CGCP analysis was performed on 338 “cases” and 338 controls, involving 482 SNPs. A total of 9,802 genotype combinations considered specific to psoriasis were identified, with the maximum number of specific combinations being 16 (4.7%). After annotating the SNPs in these combinations to their corresponding genes, 4,640 identical gene combinations were obtained, among which the maximum number of specific gene combinations was 12 (3.6%) (S7 Table).

In the second scenario, association analysis was conducted using 548,357 SNPs located outside psoriasis-associated regions, based on whole-exome sequencing data. After quality control and pruning, 570 SNPs were included in the CGCP analysis (S8 Table). A total of 63,657 genotype combinations were identified as specific to psoriasis, with the maximum observed number of specific combinations being 32 (4.1%) (S9 Table). After annotating the SNPs in these psoriasis-specific combinations to genes, 62,971 gene combinations were obtained, and the maximum number of specific gene combinations was 51 (6.5%) (S10 Table).

Based on the scale-up principle, thresholds for investigating background noise in psoriasis-specific genotype combinations and gene combinations were established. Specifically, the threshold for genotype combinations was set at 37, calculated as [(781/338) × 16], and that for gene combinations was set at 52.

- Results: The authors frequently use the term “gene combinations”. After giving it some thought, it should be clear what they mean by that. However, the term is somewhat misleading, since, after all, it is rather “combinations of variants of genes”. Therefore, I would like to recommend providing a clear definition what is meant by “gene combinations” at the first appearance of the term.

We thank the reviewer for this thoughtful suggestion. We have provided a clear definition at its first occurrence in the text, specifying that it refers to "combinations of variants of genes." All subsequent uses of the term have been revised to reflect this more precise description.

- Lines 318 and 320: Do you mean “nonsynonymous” and “synonymous” “codons” instead of “regions”?

 Thank you for correcting our mistakes. The terms should indeed be "nonsynonymous codons" and "synonymous codons."

- I would love to see a table with all genes identified, along with their function, embedded in the text. Alternatively, Supplementary Figure 3 could be embedded in the text. After all, this paper is about the causative genes for psoriasis, and not just a proof of concept.

Thank you for your valuable suggestion. We have reoptimized the original Supplementary Figure 3, embedded it in the main text, and renamed it Figure 4 in sequence. Additionally, we have added the detailed annotation information of all 134 genes to our manuscript as an supplementary file (Table S27).

- Supportive material, S26 table: The authors refer to nucleotide positions on specific chromosomes. For this to make sense, the specific release of the Human Genome Project should be indicated, also for all other tables wherein nucleotide positions are provided.

Thank you for your valuable comment. All genomic coordinates are based on the human reference genome assembly hg19 (GRCh37). We have added For each table including S26 table, we have added this information in each tables including S26.

- S24 table: Abbreviations (“BP”, “A1”, “F U”, etc.) should be defined, also for other tables.

Thanks for your advice. We have defined all abbreviations (e.g., BP, A1, F_U) in the corresponding tables (Table S1, S5, S8) as suggested.

Reviewer #4: This study aimed to identify characteristic genetic signatures of psoriasis using a novel analytical method, Causal Genotype Combination Patterns (CGCP). Applying CGCP to whole-exome data from 781 psoriasis cases and 676 controls of Chinese Han origin, the authors uncovered 620 psoriasis-specific genotype combinations converging into 134 genes, including many previously known psoriasis-associated genes. The findings suggest that population-level differences in psoriasis prevalence may be explained by varying frequencies of these genotype combinations.

The authors have conducted a comprehensive and well-designed study with detailed analyses, and the supplementary materials clearly support and explain their findings. They have also provided satisfactory responses to the reviewers’ comments. However, since the study focuses on psoriasis and involves multiple candidate genes across different pathways, it would be valuable to emphasize the potential clinical relevance and translational impact of the results. In particular, the Discussion section could briefly highlight which pathways or gene interactions are most strongly associated with psoriasis and why these pathways are biologically or clinically important.

Thank you for your constructive comment. We have revised the Discussion section (Lines 399-417) to more explicitly highlight the pathways and gene interactions most strongly associated with psoriasis pathogenesis. Specifically, we have expanded on the biological and clinical importance of these key pathways. Corresponding references (52-58) have been added to support these points.
