# Supplementary material for: A new perspective on population genetics: Deciphering the relationship between genetic variants and disease prevalence in Psoriasis
Source: PLoS One. 2026 Mar 13;21(3):e0344204. doi: 10.1371/journal.pone.0344204 (PMC12987435; doi:10.1371/journal.pone.0344204)
Supplement: Attachment — Submitted filename: Response to Reviewers-R3.docx [file pone.0344204.s041.docx]

**Responds to the academic editor’ comments:**

Dear Editor:

Thank you for your letter and for the comments of reviewers concerning our manuscript entitled “A New Perspective on Population Genetics: Deciphering the Relationship between Genetic Variants and Disease Prevalence in Psoriasis” (ID: PONE-D-24-55512). We appreciate the opportunity to address these requirements and have made the necessary revisions accordingly. Below, we respond to each point in detail.The revised manuscript, figures, and files have been uploaded to the submission system.

Reviewer #2: The authors have made many corrections and addressed some of my concerns. However, I still urge the authors to read through the manuscript carefully, as there are still many inconsistencies in the notations.
We would like to express our sincere gratitude for your insightful and constructive comments. We apologize for the oversights in the original manuscript. We have addressed each of your suggestions point by point and have refined the details throughout the text to enhance the overall clarity and readability of the manuscript.

I will start with some new comments not regarding notations:
In line 255: I presume the authors wish to say p-value instead of level of significance? Also, since a large italicised P was used to define prevalence, using a large P to define p-value can be confusing. Simply saying “a p-value of 0.008” should be sufficient. Or else a small p is more commonly used for p-values.
Thanks for your advice. We have made the revision at the corresponding location and have also corrected similar errors throughout the manuscript to ensure consistency.

In line 272: Type I and Type II are more commonly used terms.
Thank you for pointing out the inappropriate expressions. We have revised the text accordingly in the manuscript.

In line 273: I can’t seems to find it, but are the details of the three models described somewhere? If so, they should be referred, if not, they should be somewhere.

Making links, references, tables, figures and sections clickable when referred to so it jumps directly to the referred component will make the reading experience much better. This needn’t apply to separate documents, for example supplements.
Thank you for your suggestion. We sincerely apologize for the oversight, which may have caused some confusion for the readers. We have now inserted a hyperlink at this location to direct readers to the comprehensive details regarding these three models.

Notations:
Again, these are only a few examples, please also fix anything else that remain.

We sincerely appreciate the reviewers' positive suggestions, which have greatly enhanced the quality of this study. We have carefully corrected the errors and updated the text to present a more rigorous study.

In line 37, but also in other cases, choose whether there should be a spacing before and after an operator.
Thank you for your suggestion. We have standardized the formatting of mathematical operators throughout the entire manuscript to ensure consistency.

In the same line, the confidence interval is usually written in the form [a,b] instead of a-b.
We have revised the equation and the confidence interval format according to your suggestions. The revised version is as follows: y=61.72x+0.48 (95% CI [21.60, 101.84]). We have also ensured that this [a, b] format for CIs is used consistently throughout the manuscript.

In line 86, I believe the reference numbers are in a smaller font.

Thank you for the suggestion. We have revised all the references to comply with the journal's required format.

For equation 1, simply calling it genotype combination patterns instead of No. of genotype combination patterns could incite confusion. It might be easier to define it as something simpler, for example, calling it N, in a previous sentence.
Thank you for this excellent suggestion. We have revised the description of Equation 1 to ensure clarity and to prevent any potential misunderstanding by the readers.

Equation number should be flushed right to avoid confusion with the equation itself.

For equation 2, it’s more standard to define the population genotype frequency as F_i, and its estimator given in the equation as \hat{F_i}. It should also be made clear what ‘i’ represents here.

For equation 3, ‘i’ should not be in italic, it should be made clear that the product is over ‘i’.

Following your suggestion, all equation numbers have been moved to the far right margin to clearly distinguish them from the mathematical expressions.

We have updated F_i to $\hat{F}$i in Equation (2) and throughout the subsequent text to denote that it is an estimator. We have also added a definition for the index: "... Where the subscript i represents the index of the i-th genotype.

We have corrected the formatting in Equation (3).The index ‘i’ has been changed from italic (*i*) to Roman font (i) as requested. The product notation has been updated to explicitly include the lower and upper limits (i=1to r), making it clear that the product is taken over the index i.

In line 163: ‘were described in the manuscript’, mention where.
Thanks for your advice. The details of these simulations and the results were described in the supplementary file called .Supplementary _computer program simulation.

In line 177: inconsistent notation of f
We apologize for the oversight. We have unified the notation of "f" throughout the entire manuscript to ensure consistency.

In line 201: inconsistent notation for decimal
Thank you for pointing this out. This has been corrected.

In line 207: I believe it should be bold font like other subsections, the bold fonts for the subsections are also not very obvious.
We appreciate the reviewer's suggestion. The subheading in line 207 has been bolded. Additionally, we have updated the formatting of all subheadings in the Methods and Results sections to a more prominent bold font to improve visibility and readability.

In line 227: inconsistent product sign
We apologize for the oversight. This has been corrected.

The reference format is also not consistent.

We apologize for the inconsistent formatting of the references. We have thoroughly reviewed the entire manuscript and standardized all in-text citations and the reference list to strictly adhere to the journal's formatting guidelines.
